# Distance, accessibility and costs. Decision-making during childbirth in rural Sierra Leone: A qualitative study

**DOI:** 10.1371/journal.pone.0188280

**Published:** 2018-02-20

**Authors:** Laura Treacy, Håkon A. Bolkan, Mette Sagbakken

**Affiliations:** 1 Department of Community Medicine, Institute of Health and Society, University of Oslo, Oslo, Norway; 2 Institute of Cancer Research and Molecular Medicine, Norwegian University of Science and Technology (NTNU), Trondheim, Norway; 3 Faculty of Health Sciences, Department of Nursing and Health Promotion, Oslo & Akerhus University College, Oslo, Norway; 4 The Norwegian Centre for Migration and Minority Health (NAKMI) Gullhaugveien 1–3, Oslo, Norway; Helsingin Yliopisto, FINLAND

## Abstract

**Background:**

Sierra Leone has one of the highest maternal mortality ratios in the world. Efforts to reduce maternal mortality have included initiatives to encourage more women to deliver at health facilities. Despite the introduction of the free health care initiative for pregnant women, many women still continue to deliver at home, with few having access to a skilled birth attendant. In addition, inequalities between rural and urban areas in accessing and utilising health facilities persist. Further insight into how and why women make decisions around childbirth will help guide future plans and initiatives in improving maternal health in Sierra Leone. The objective of this study was to explore the perceptions and decision-making processes of women and their communities during childbirth in rural Sierra Leone.

**Methods and findings:**

Data were collected through seven focus group discussions and 22 in-depth interviews with recently pregnant women and their community members in two rural villages. Data were analysed using systematic text condensation. Findings revealed that decision-making processes during childbirth are dynamic, intricate and need to be understood within the broader social context that they take place. Factors such as distance and lack of transport, perceived negative behaviour of hospital staff, direct and indirect financial obstacles, as well as the position of women in society all interact and influence how and what decisions are made.

**Conclusions:**

Pregnant women face multiple interacting vulnerabilities that influence their healthcare-seeking decisions during pregnancy and childbirth. Future initiatives to improve access and utilisation of safe healthcare services for pregnant women need to be based on adequate knowledge of structural constraints and health inequities that affect women in rural Sierra Leone.

## Background

Maternal mortality and morbidity remain international public health problems. In 2013 it was estimated that there were 292,982 maternal deaths globally, with 99% of these occurring in developing countries [1–3]. Global efforts to improve maternal health have included attempts to increase the proportion of women giving birth with a skilled attendant [4–6]. This often goes hand in hand with women attending antenatal appointments and delivering at a health facility [7], which in turn rely on an effectively functioning health system [6, 8–10].

Maternal mortality in Sierra Leone remains persistently high with a ratio of 622.6 per 100,000 live births in 2013 [1], despite measures to increase the number of women delivering at a health facility [11, 12]. These measures include informal bylaws that are thought to encourage ‘facility care’ through fining women and their communities if they do not attend the hospital for antenatal care or fail to give birth at a facility [11]. Prohibitive costs have been shown to be a major factor in preventing women accessing health facilities during childbirth in Sierra Leone [11, 13, 14]. In 2010 the government introduced the Free Health Care Initiative (FHCI) for pregnant women, lactating mothers, and children under the age of 5. According to the 2013 Sierra Leone Demographic Health Survey (DHS) 97% of women had attended at least one antenatal care appointment with a skilled provider during their last pregnancy [15]. It should be noted that this high percentage does not necessarily indicate anything about the quality of care received. Despite this high percentage of women accessing antenatal care and regardless of the FHCI, and introduction of informal bylaws, 45.6% of women did not deliver at a health facility in 2013 [15]. This suggests that the removal of user fees alone are not enough to encourage women to utilise health facilities during childbirth, which is similar to experiences in other countries [16–18]. The majority of Sierra Leone’s population live in rural areas [19]. The 2013 Sierra Leone DHS found that utilisation of health facilities to deliver differed between urban and rural areas: 68.1% in urban areas and 49.7% in rural areas [15]. These inequalities persisted for delivery with a skilled attendant, at 78.9% delivering with a skilled attendant in urban areas and 53.2% in rural areas [15]. These statistics illustrate how location of where women live can impact upon their use of health facilities and skilled birth attendants during childbirth, and that focus on women living rurally is important.

A review of the literature indicates that understanding how and why women and their communities make decisions during childbirth is crucial when creating and implementing initiatives aimed at reducing maternal mortality and morbidity [20–24]. This paper explores who and what influences the decisions made by women and their communities within rural areas of Sierra Leone; and how the position of women in society and structural constraints they experience impact upon these processes.

## Methods

### Study design and study area

This qualitative study involved focus group discussions (FGDs) and individual and group in-depth interviews (IDIs) with recently pregnant women and members of their communities. We used partly homogenous sampling for the FGDs, and purposeful sampling to obtain maximum variation for the IDIs [25]. Themes and topics discussed during interviews and conversations were partly informed by literature and recent initiatives and partly by participants introducing different themes throughout the fieldwork.

The study took place between August and December 2013, in Tonkolili District. This district was purposively chosen as statistics showed that in 2013 fewer women gave birth in a health facility in Tonkolili District compared to the national average [15]. Two rural villages in the Kholifa Chiefdom (La-lenken section) were included to ensure variation concerning geographical site, population size and socio-demographic variables. Inhabitants in both villages used Masanga Hospital for their main institutional needs. This NGO-supported governmental hospital operates as both a Primary Health Unit and a hospital, providing comprehensive emergency obstetric and newborn care 24 hours a day, seven days a week.

The first village chosen had two potential routes to access Masanga Hospital. One along a narrow bush road, accessible only by foot, that took approximately 30 minutes at a moderate walking pace, and included three rivers to wade through (higher water levels during rainy season, and more like streams during the dry season). The other option was along a ‘main road’ on a motorbike, a more convoluted route, which took about 45 minutes with no big rivers to cross but very poorly maintained roads.

The second village had one road to access Masanga hospital, which was only accessible by foot or motorbike. Occasionally a car could be seen driving along this route, but would often become stuck along the way. This road would take at least 45 minutes by motorbike and included four rivers and a number of small streams to cross between the village and hospital. Two bridges were temporary and crudely built, so passengers would often disembark and cross by foot. The rivers often flooded during rainy season and people would wade across with water up to their necks.

The research team consisted of the first author (LT) and a research assistant who was originally from La-lenken section and spoke both Temne (the local language) and English fluently.

### Participants and sampling strategy

The research team met with village chiefs, a youth leader and three Traditional Birth Attendants (TBA) in order to introduce the project and explain the criteria for participant selection. One of the village chiefs and the TBAs later participated in IDIs. Potential participants for focus groups were identified with assistance from the TBAs and village chiefs. Initially three FGDs, formed of 4–9 participants, were facilitated at each village. The different groups were: 1) women who had been pregnant within the last year; 2) older women who had been involved in a delivery or had a close family member in the village who had been pregnant within the last year and; 3) men who had a close family member in the village who had been pregnant within the last year. A final FGD was conducted at the end of the data collection period with recently pregnant women to see if new themes would emerge and to increase validity of the initial analysis of findings. Each FGD lasted between 30 and 90 minutes and was facilitated in quiet, sheltered locations on the peripheries of each village in order to limit interruptions and increase privacy.

Ten individual in-depth interviews (IDIs) were conducted with women who had been pregnant within the last year (five in each village). Participants from the FGDs who were thought to have particular in-depth, pertinent knowledge on the topic were also asked to participate in an IDI. Other potential participants were approached whilst the research team walked around the villages, speaking with different community members. The research team used purposeful sampling to obtain maximum variation for the IDIs, seeking out different participants according to appearance/dress, age, or where in the village they lived. Some factors such as appearance/dress were specifically chosen in an attempt to include a spectrum of participants with different socioeconomic statuses. The research assistant provided advice on how the different clothes/material worn could indicate different types of socio-economic statuses. In addition, a snowball sampling approach was utilised as TBAs or other well-known women in the villages identified specific potentially ‘information rich’ participants who the research team may not have been able to identify easily. These were, for example, those who had grown up in a different area of Sierra Leone and had moved to the village (usually when they got married) and therefore did not have their own immediate family members nearby during pregnancy and childbirth, or those who had experienced a complicated birth. Each IDI lasted between 45 to 120 minutes and the participants themselves chose the locations.

Individual and group IDIs were also held with additional informants that during the fieldwork were found to hold important information including midwives and community health officers (CHOs) based at Masanga Hospital, a village chief, three TBAs, and a group of motorbike drivers (12 respondents in total). The motorbike drivers often escorted pregnant women from the villages to Masanga Hospital and their experiences and insights arose as potentially informative. Five informal discussions with informants including representatives from the local radio station, and national and international non-governmental organisations working in women’s health were conducted throughout the data collection period. Deeper insight and understandings into the local discourses around women’s health were gained through these discussions. Locations for interviews with the additional informants were chosen by the participants themselves, and took place at their work places, in the village or near their homes. In total 61 participants were formally included in the study: 22 participated in IDIs and 42 in FGDs (three participants were part of a FGD as well as an IDI).

Re-interviews were conducted with seven participants at least one additional time in order to clarify unclear discussions or topics, or to discuss emerging themes highlighted in previous interviews.

### Data collection and data analysis

The FGDs and IDIs were led by LT using thematic guides, with the research assistant translating between English and Temne. Questions and shorter answers were translated verbatim, whilst longer descriptions or discussions were summarised. All FGDs/IDIs were audio-recorded. The main themes initially discussed were: experience of pregnancy and labour; who was involved during the delivery process and their roles; differences between delivering in the village and the health facility; risks or problems during pregnancy and childbirth; knowledge of the FHCI. Different themes or significant topics identified during the FGDs subsequently helped inform the IDIs. Throughout the fieldwork, as new themes emerged, interview and discussion guides were subsequently adjusted.

LT wrote summaries and reflections after each discussion/interview and transcribed all recordings immediately. Together, the research team re-listened to these audio-recordings and read the summaries and descriptions. Any summarised sections were expanded at this point so that participants’ original words and descriptions were transcribed verbatim. Re-listening to the audio-recordings together enabled any misunderstandings or mistakes to be identified and corrected. Further confidence in the quality of the interpretation of data, including both translation and cultural meanings behind discourses, was gained as a number of audio-recordings were re-translated by an independent person regularly during the fieldwork.

Preliminary analysis took place throughout the fieldwork period, with both LT and the research assistant taking active roles. Daily reflections, a field diary and conceptual maps all assisted to identify emerging themes, and modify the interview guides. Systematic text condensation, as developed by Malterud [26], was used to analyse data concerning factors that affected the perceptions and decision-making processes during childbirth. The analysis followed these steps: (i) all material re-read to get an overall impression; (ii) identifying and sorting the material into codes, (iii) condensing the codes to themes, and (iv) analysing these themes so that descriptions that reflected significant factors were formed.

The qualitative analysis software NVivo10 was used to organise the data into codes and themes, and in this way gain an overview of the different perspectives, nuances and clear patterns as eventually described in the findings. Codes were initially formed with suggestions from previously read literature as well as topics that emerged during the preliminary analysis within the field. Codes and categories were continuously reflected upon, adapted, subgroups formed, changed and merged as appropriate. Previously coded transcripts were re-visited with any newly adapted codes.

Finally each transcript, with all the identified codes and categories, were printed and re-read. The analysis was summarized and accounted for in an analytical document that was used as a basis for writing and revision of the manuscript, and that LT and MS worked on.

### Ethical considerations

The study gained approval from the Sierra Leone Ethics and Scientific Review Committee and Norwegian Social Science Data Services (NSD). Approval was sought from the Regional Committees for Medical and Health Research Ethics in Norway, but it was not necessary as they considered the study to lie outside of the Act on medical and health research. La-lenken section and village chiefs gave approval to conduct the study in their villages. Informed written or finger print (depending upon the participants literacy level) consent was obtained from all participants, and they were informed that they could withdraw from the study at any time, for any reason, without negative consequences. Names and other identifying-information were removed from transcriptions and analyses to ensure confidentiality. The research team provided written information about the project to each participant. Those who were unable to read were verbally informed about the research project. The Sierra Leone Ethics and Scientific Review Committee and NSD approved this consent procedure.

## Results

The findings reported in this paper are from a wider study exploring the broader perceptions and decision-making processes related to childbirth in Sierra Leone. This manuscript focuses on three different themes, with “money” often featuring as a significant factor. One theme is distance and accessibility, which includes the subthemes long distances and impassable rivers as well as the discomfort, cost and availability of transport. Another theme is healthcare costs and staff, which include the subthemes direct and indirect costs of healthcare, and expectations regarding the treatment by staff. The last theme is the role of men and the extended family. Findings relating to themes such as home delivery being the norm, perceptions of bodily symptoms and the influence role of religion are presented in another article [27].

### Distance, accessibility and costs

#### Long distances and impassable rivers

Most rural villages in this area are only accessible by off-road vehicles or motorbikes due to the poor road conditions, especially in the rainy season. The distance to the hospital as well as lack of accessible and affordable vehicles were significant barriers when attempting to go to the hospital to deliver. Many women were reluctant to walk so far; did not want to walk alone; and were fearful of giving birth along the bush road, a situation that many had already experienced. One woman shared her experience of trying to get to the hospital along the bush road:

*“(…) as soon as I felt the labour pain I decided to go to the hospital to deliver there*. *But since I started to give birth*, *I have never been to the hospital yet*, *although I have (had) the plan to go there*. *I have given birth to 3 children along the bush road when I was going to Masanga Hospital*.*”* (IDI 22)

This woman, along with others, had tried on many occasions to get to the hospital, but had either had to turn back or had delivered along the bush road. These types of experiences illustrate how final decisions during childbirth may be pragmatic and ad hoc, influenced by the immediate circumstances the woman finds herself in. In a majority of the cases, the inaccessibility of safe, affordable transport from the home to the hospital meant that women had to walk along the bush road. However, the distance from the village to the hospital was so far that often it was not considered an option, or as illustrated in the quote–many would end up giving birth on their way.

In situations where women did attempt to walk along the bush road, they were accompanied by a number of female relatives or neighbours. Occasionally women would be carried by the village men in a hammock, but this mode was reliant on enough men being willing and able to carry her. The difficulties with distance and lack of transport were also highlighted by one of the CHOs describing a case at the hospital:

*“They brought the woman in a hammock*. *The woman was in labour (…) there is no access for a vehicle to go to that village*, *and she had to be carried in the hammock*. *Strong men had to carry her*, *and they rushed with her here*. *By the time they arrived it was too late (…) it was too late for the baby to survive”*. (CHO 53)

This example, along with other similar stories, illustrates how long distances and lack of transport limits communities’ ability to access health services, and in turn directly impacts upon infant and maternal mortality.

Another problem the women encountered was crossing the many rivers, especially in the rainy season, as pointed to by one of the CHOs:

*“There is a river in this place (…) people don't have access to cross this river*. *Suppose you are a pregnant woman*, *how can this woman cross this water here?”* (CHO 54)

The CHO was referring specifically to the rainy season when many of the rivers are impassable, or require women to wade up to their necks to get across. Even if the rivers do have a bridge, many of them are temporary and dangerous to cross. Another participant recounted a story about a woman from a nearby village:

*“…They took the patient to the hospital*, *and at that time there was no baby and the pregnant (woman) became dead*. *Because they wasted too much time*.*”* (IDI 13)

He described how the delay in reaching the hospital during childbirth resulted in both the woman and the baby dying. This reality of babies and women in labour dying, or at risk of dying, due to not arriving at the hospital in time, was discussed by the CHOs, TBAs and midwives, and indirectly illustrated through the many examples mentioned by the different respondents.

#### Transport–discomfort, cost and availability

As stated above, many of the roads to and from the hospital were narrow dirt tracks and were thus, usually only accessible by motorbike. Obvious discomfort of being on a motorbike whilst in labour, as well as the potential danger of the narrow and slippery roads, were factors many participants spoke about when deciding to travel by motorbike or not. The motorbike drivers themselves spoke about the difficulties in taking a pregnant woman as a passenger:

*“If a pregnant woman told me to go with her*, *I was afraid of the pregnant woman (…) to carry her*. *If I carry a pregnant woman on my motorbike*, *I am afraid to take speed*. *I need to take my time because she is in pain*. *That is why I find it so difficult to carry the pregnant woman on my motorbike*.*”* (FGD 56)

The motorbike drivers linked the need to drive extra carefully with the fact that they felt responsible for two lives when carrying a pregnant woman. The motorbike drivers also risked losing money when taking a pregnant woman unexpectedly during an emergency. They spoke of leaving their current (paying) passengers, in order to carry the woman in labour, but not feeling it appropriate to discuss cost of the fare in an emergency situation.

The women, on the other hand, claimed that payment for the motorbike could not be credited, and would usually be paid for up front. In addition to the cost of carrying the mother, they would also have to pay for the carer and often the TBA to accompany her. Even if a woman wanted to, and had the financial means to take a motorbike, availability was not always guaranteed, especially at night. Availability of a willing driver was also not certain, as discussed in one of the female FGDs:

*“Some of the motoboys (*locally used name for motorbike drivers) *will be afraid*, *they say that they won't go with the pregnant woman to the hospital when they are in labour*. *They think that the pregnant woman will give birth along the way (…) They don't want to see where a woman gives birth (…) he is afraid to take me along*, *so I can't force him”*. (FGD 26)

This additional concern was related to all motorbike drivers in this area being male, and since men are not permitted to view a woman give birth, neither the pregnant woman, nor her motorbike driver want to be in the situation where he may witness her give birth en route to the hospital.

### Healthcare costs and staff

#### Direct costs in accessing health care

In addition to transport costs, direct costs related to paying to ensure “good treatment” were also described as a barrier to accessing health services at the hospital. The implication of the need for money when seeking care at the hospital was illustrated in the discussion in one of the male FGDs:

(34) The other people who have money gave a lot of money to the nurses. You will see they give a lot of treatment to that wife.

(35 interrupting)… a lot of medicines.

(34)… while you who does not have money, they will only give you paracetamol. For the other people, if you take your wife to the hospital, without giving extra money, they won't take care of your wife.

(31) The nurse told us ‘this is our area where we can eat, so you need to give us something’.

*(32) We here in Africa (…) everything is very costly if you don't have money*.*”* (FGD 31, 32, 34, 35)

As illustrated in the quote and confirmed by key informants, health personnel working in remote health centres regularly do not get paid on time or if they do get paid it can be very little. Therefore, many are reliant upon some form of payment from patients in order to provide for themselves and their own family.

The requirement to have ‘money at hand’ to pay for services at the hospital was often described as a need to show appreciation. Thereby, recognising and appreciating the skills and dedication of the hospital staff in a way that the government usually does not. Both service users and health care providers, as illustrated in these two quotes, discussed this distinction:

*“It doesn’t mean that when I gave birth in the hospital the nurses took money from me*. *But when somebody helps you to give birth safely*, *you need to do good things to her*.*”* (IDI 8)

*“In life different people take things differently*. *And some will say I am doing this for you*, *it's free*, *you are not paying anything*, *although I am being paid; at least a sign of appreciation*, *you need to appreciate*.*”* (CHO 54)

The need to show your appreciation was also acknowledged when receiving care by the TBAs or the older women, but the payment could be in the form of services such as washing clothes or fetching firewood, or could be negotiated or credited, and thus paid at a later stage. One of the women who delivered at home in the village with assistance from a local trader, described such a situation:

*“For us here*, *the man who gave me the injection*, *we only credit to him to inject me (…)*. *The man was charging me 20*,*000Le (about $5)*, *we talked a price*, *and later he told me to give him 15*,*000Le*. *He gave me three injections together with the medication tablets*. *That was the time I gave birth*.*”* (IDI 24)

This woman shared her experience of receiving biomedical medicine in the form of tablets and injections from a trader in the village. These tablets and injections were thought to speed up labour and reduce pain. Obtaining biomedical medicine this way was deemed easier as the trader with the medication would come to the labouring woman, rather than the woman having to travel to the hospital. Participants in the second village also discussed the services of a trader providing advice on the progress of labour and administering biomedical medicine. The trader was described as an “old granddad with experience in childbirth” that lived in a nearby village, offering his services mainly to earn money.

#### Indirect costs of healthcare

In addition to the direct costs of delivering at the hospital, indirect costs were another factor that could influence where women gave birth. Some participants spoke about a perceived social expectation of buying items for their newborn, such as a cloth to wrap the baby in. If they were unable to afford these items they would feel ashamed, and therefore they would not want to attend the hospital to deliver.

Women who were expected to stay as ‘waiting mothers’ (residential facilities where women who live remotely can wait before giving birth at a hospital or health centre) often bore the brunt of indirect costs. Staying in the hospital meant that they were no longer earning money through business, mining or farming. In addition, for every woman admitted, a carer had to be with her: to wash and cook for her since these tasks were not part of the nursing role at the hospital. This carer was also not able to carry out her own usual tasks and responsibilities. Husbands or other family members were then expected to care for children at home, again limiting their ability to work or do their farming. In that way, accessing services at the hospital, the daily life as well as the household economy of several families, were heavily influenced. One woman described how she refused to be admitted as a waiting mother at the hospital:

*“(…) the nurse told me I needed to be admitted but I refused*, *just because there is nobody taking care of my children at home*.*”* (IDI 1)

This woman, along with several others having similar examples, did not want to stay as a waiting mother because this would involve abandoning her responsibilities at home, as well as causing an additional burden when trying to find food whilst staying in the hospital.

#### Expectations regarding the treatment by staff

Another potential barrier to attend the hospital was the perceived potential behaviour or reception by the staff. Some of the participants spoke about the lack of respect they expected to receive at the hospital, and some also spoke about a specific type of ‘advice’ that had been given by the nurses at the antenatal clinic, as explained by one woman during a focus group:

*“The nurse told me to go to the hospital to give birth there*. *(She said) if you force yourself to give birth in the village and unfortunately a problem occurs (…) don’t then come here (to the hospital)”* (FGD 24)

The statement above relates to the fact that many women believed that if they tried to deliver at home, but then a problem arose, the nurses may not treat them, or would treat them badly because they delayed attending in the first place. Another woman, who had delivered at the hospital, shared her experience of being alone because her mother had not been allowed to be in the delivery room with her. She emphasised the importance of having her family there, to provide care and support, and thus giving birth at the hospital was not perceived as a good experience.

There was also a perception that multigravida women would be treated with less patience than primigravida women, a notion explained by one man:

*“The nurses will say ‘ Oh don’t come and cause noise for us*. *We thought this was the first born*, *you have given birth for four children*, *now you want to cause noise for us*, *don’t shout here’… that is why people are afraid to go to the hospital”*. (IDI 13)

He was describing that many feel that if you have delivered before, you are expected to give birth easily and quietly during your subsequent deliveries. In the hospital, this expectation is perceived to result in impatience or lack of care from the staff. Other participants, again many who had in fact never attended the hospital to deliver, shared this concern about multigravida women not being treated as well. This suggests that hospital staff may overlook that each individual childbirth experience can vary greatly from pregnancy to pregnancy. This is particularly the case if their previous deliveries were at home in familiar surroundings and they find themselves delivering in a hospital for the first time.

### The role of men and the extended family

Most participants agreed that husbands have the right to tell their wives what to do, as he had married her and was now responsible for her. This right often translated to ‘permission’ needing to be sought from the husband before a decision could be made to go to the hospital. The health workers also discussed having to acquire the husband’s consent before commencing treatment. One CHO described a recent situation at the hospital where a woman required a caesarean section, but the staff were reluctant to operate before they had the husband’s permission:

*“They were very*, *very reluctant*, *because the husband was saying that ‘if somebody does a C-section on my wife without my permission I will take that person to court’*.*”* (CHO 53)

Waiting to get the husband’s permission, especially if he was not in the vicinity of the hospital, could be another delay that prevented the pregnant woman receiving the appropriate and timely care that she needed. The midwives, however, emphasised that the staff would not wait for permission if they had to act immediately to save the woman’s life.

It was also deemed the responsibility of the husband to find and provide money for transport to attend the antenatal clinics or access the hospital during labour. One mother described a situation when her husband had not provided her with money to attend the antenatal clinic. She was, however, able to earn some money herself through selling food from her farming, and she used this to attend the clinic. This example illustrates that increased financial independence allow some women more autonomy in the decision-making process.

Childbirth is often referred to as “women’s business” in Sierra Leone and only women who have already given birth themselves are allowed to witness it. In the focus group discussions older women expressed that it was not men’s responsibility to ‘know about’ childbirth: such as understand what happens, or participate in the birthing process. A husband would often wait outside the home where his wife was delivering and rely on female family members to share information about the progress of the birth. This means that men, who do influence the decision-making processes during childbirth, in fact have little knowledge on which to base their decisions on. One participant explained that men usually prefer their wives to give birth in the hospital, but they do not know why most women actually end up giving birth in the village:

*“As for us who marry the wives…*.*we really want them to go to the hospital to give birth*. *But some will not*. *We don’t know the reason why they decide to give birth here*.*”* (IDI 13)

This quote illustrates how men, despite being ‘decision-makers’ in the community, do not seem to have deep insight into why or how decisions are actually made.

Some men went away to other provinces or areas regularly to find work, usually mining, thus being absent during the time of delivery was not uncommon in the villages. These women, along with unwed, widowed or single women, were thus often reliant on the extended family to help them. Some women stated that they had actively decided to go and stay with their own mothers because they wanted to be with someone familiar, who had experience in delivering, and would assist with the baby after the birth. This was particularly the case if the woman moved with her husband to a new area and did not have a familiar social network in place.

## Discussion

The findings from this study demonstrate that decision-making processes in general are dynamic and flexible and influenced by the many interrelated factors as described above. Ultimately, the final decisions may not be the preferential choice, but can be pragmatic, based on the situation and conditions the women and their communities find themselves in at that point in time.

### Poverty

Prior to the introduction of the FHCI in Sierra Leone, many studies documented the prohibitive costs of services as one of the main factors in preventing people accessing care in the hospitals during childbirth [11, 13, 14, 28]. However, direct or immediate cost of services is only one of many financial barriers that can prevent people accessing formal healthcare services. Findings from this current study illustrate that other types of poverty-related barriers are significantly influential in the decision-making processes during childbirth for many people living in rural Sierra Leone. This is in keeping with a number of other studies in low resource settings [7, 16, 29–32].

In this study participants described the importance of having ‘money at hand’ in order to access quality-care at the hospital and ensure good and empathetic treatment during delivery. A study conducted before the FHCI in Sierra Leone also reported a perceived need to pay staff in the health facilities, to ensure ‘good treatment’ [11]. In this current study, giving money for services was often described as a ‘sign of appreciation’ rather than direct payment. Regardless of whether it was seen as payment or a gift, the perception persists that money is needed in order to access treatment at the hospital.

The need to show a ‘sign of appreciation’ for services was also apparent in the villages, especially since the FHCI does not extend to services provided by TBAs, who are therefore dependent on payments from the women they help [24]. In contrast to the hospital where payment was provided in hard cash, payment in the village could be ‘in kind’ e.g. collecting wood or cooking a meal, or credited and paid over time. This meant that families would not necessarily have their financial status or level of poverty so evidently exposed when paying for services in the village.

There were a number of indirect costs associated with delivering at the hospital, such as loss of potential income, and the need to ensure that someone was able to carry out the woman’s additional domestic and farming responsibilities. Another phenomenon that was highlighted in the findings was that of social expectations around birth [29]. Women in this study spoke about the expectation to provide a clean or new lapa (the cloth women typically wear as a skirt or dress) for their newborn. In the village, family and friends could wash the pregnant woman’s cloth, ensuring she had a clean lapa to wrap her newborn in, negating the need to actually buy a new one. In the hospital, where washing clothes is not as easy, they would feel a pressure to buy a new lapa. Not having money at hand to buy a new one could identify a woman as being poor.

A position of poverty can imply that an individual is perceived as representing a lower, less powerful level of society, which in turn can bring with it neglect or lack of respect from those around, in this case the hospital staff. Medical anthropologist James Trostle [33] argues that social institutions, and subsequently health personnel working in these institutions, are structural expressions of the power-imbalance that exists between social classes. As a result, existing power structures in the society at large are exercised and maintained in face-to-face interactions between health personnel and their patients [33]. According to the different respondents in this study, poor women were particularly prone to negative attitudes and received less care from health personnel in the hospitals. Power is not equally distributed between social classes and neither is the power to resist [34]. Being a poor, rural woman in this setting may cause a state of powerlessness that makes it very difficult to oppose such unequal treatment.

In addition, the hospital is a public space, and therefore, what happens in the hospital can rapidly become known by a wider audience. Any shame associated with not meeting social expectations in the hospital, and thus being identified as in a position of poverty, becomes publicly known. This creates a double burden of being poor that can further inhibit the ability to access healthcare services [35]. The fear of being shamed, and subsequently laughed at for not meeting these social expectations, was ultimately enough to motivate some women to deliver at home rather than in the hospital. These findings are similar to a study in rural Tanzania where attending the hospital wearing your best clothes and carrying items for delivery, such as gloves, was socially expected [36]. To arrive in poor or dirty clothes increased the risk that staff would abuse or neglect the women. Again, this fear of being publically shamed for not attending the hospital with the correct clothes or delivery items could be enough to convince the women to deliver at home [29, 36].

The variety of direct and indirect costs associated with living in a rural area was another barrier facing the communities in our study. The distance from these rural villages to the hospital, poor road conditions, inaccessibility particularly during rainy season, and the lack of safe, affordable and reliable transport, were all factors affecting the decision-making process in this study. All of these “every day inequalities” faced by the communities in this study can be described as a form of “structural violence” [37]. Paul Farmer describes structural violence as systematic ways in which, often invisible, social structures harm or in other ways disadvantage individuals. As shown in this study, unequal distribution of resources, money and power imply that safe and affordable health services often remain inaccessible to women in poor, rural communities. These types of structural barriers, such as poor road conditions, lack of available ambulances and shortages of equipment and medical supplies have been discussed in previous studies conducted in other parts of Sierra Leone [11, 13, 14] as well as in studies from other low resource settings [7, 16, 21, 29, 30, 38–40].

### Women’s position in the home & society

Sierra Leone is a patriarchal system, whereby men are the head of the household and responsible for main decision-making [11, 41]. Since men are dominant with regards to household matters in Sierra Leone, they also have central and controlling positions in the wider sphere of community and political discourses. A number of actions and policies have been implemented to improve gender equality in the country but large inequality still remains [42]. In 2011 it was estimated that only 9.5% of females in Sierra Leone gained a secondary school education, whereas 20.4% of males gained secondary education [43]. Other estimates suggest that 85% of women remain uneducated [42]. This lack of education, along with exclusion from public positions, may negatively impact upon women’s abilities to negotiate and participate in household decisions, including decisions regarding healthcare. This means that men have the potential to be highly influential in the decision-making processes during childbirth in rural Sierra Leone.

In this study childbirth was viewed as “women’s business”, meaning that only women need to know what happens during childbirth i.e. excluding men and children. Men’s main responsibilities during pregnancy and childbirth were considered as providing money and acquiring transport if either were needed. These findings are similar to studies conducted in other parts of Sierra Leone and Ethiopia [11, 23]. Predominantly, men were not found to be actively involved in the decision-making process during childbirth. However, their control of assets and finances, as well as their position in society would often ensure some degree of power over the decisions made. Other studies have also documented this position of men being in control of resources and thereby asserting power over a large part of the decision-making process [11, 30]. A systematic review in 2013 found that women who had access to their own money, and therefore were not solely reliant on their husband for financial assistance, demonstrated more empowerment and autonomy during the decision-making process during childbirth [29].

In addition to the control over finances, women in rural Sierra Leone are often required to gain permission from their husbands before they can attend hospital or access certain treatment [11, 13], a situation found in other parts of sub-Saharan Africa and Asia [29, 30]. This need to obtain permission can be inhibitory for women accessing appropriate and timely healthcare. The combination of limited understanding or knowledge about the birthing process, with power over providing permission and finances, can result in men making potentially harmful decisions.

### Amalgamation of vulnerabilities

The Human Right to Health is protected by many documents [44, 45]. For women, this includes the right to reproductive health care. In order to utilise this right women need to be able to access health services, and governments need to address barriers (financial, structural and social) that prevent women achieving this human right. Women in rural Sierra Leone face multiple health vulnerabilities, which often result in them being unable to access their right to health.

Intersectionality, as originally discussed by Crenshaw [46], explores how the different aspects of race, gender and class interact and work concurrently to produce inequality. Intersectionality can be useful when discussing how the many different facets of ‘being a woman in rural Sierra Leone’ can simultaneously impact, overlap, augment and create inequalities. The different identities associated with being a woman in rural Sierra Leone can influence what options or decisions are available to her, the woman’s ability to make decisions, and which decision her and her community finally make. These decisions will ultimately impact upon the final health outcomes for these pregnant women.

For a poor woman in rural Sierra Leone, her gender will have played a part in her decision-making processes from the minute that she was born a girl, and will continuously affect the process throughout her life. Gender will have dictated her educational opportunities [42, 43], potentially her ability to choose if or when she would have her own children, her autonomy and voice, or lack of, within her home, the wider community and on a national level [47]. Lack of education will mean that she will have reduced opportunity to paid employment. This lack of money will further reduce her power within the household; potentially further limiting her voice within household decisions, strengthening her husband’s ‘permission-giving’ role, so that she has little control over the decisions made regarding her own health. The perceived requirement of ‘money at hand’ to access good treatment at the hospital means that women’s positions of poverty further reduces their ability to access health services.

Being born in rural Sierra Leone means that geographically a woman is further away from the hospital in order to access formal-healthcare. It takes longer to get to the hospital for check-up appointments or whilst in labour. More time away from the home means reduced ability for income-generating activities, the woman becomes dependent upon others to care for her children, farm her crops or attend to other domestic duties. She requires cash to pay for a motorbike to take her to the hospital, and the further they need to drive, the more petrol the woman must pay for. She already has limited resources and to access the formal healthcare at the hospital ‘costs’ her more than it potentially would, say, a man, living in a main town or city, closer to a hospital. The many different identities associated with being a poor woman in rural Sierra Leone, work together to create these large health inequities.

Women’s social status, as dictated by their gender and level of poverty systematically denies them access to certain resources such as education and health services [37]. This becomes a vicious circle as reduced access to health services increases their risk of ill health, further limiting employment opportunities and ability to earn money, another example of structural violence [35]. Reduced economic power reinforces gender inequalities that are likely to be present. As discussed by Michael Marmot in his book ‘The Health Gap: The Challenge of an Unequal World’ [48], inequality and poverty are significantly disempowering. This disempowerment further limits any autonomy women may hold over decisions that directly affect their lives, and their health, such as decisions made during childbirth regarding *who* assists them, when and where.

The failings in infrastructure that the women in these poor, rural communities face are outside of their individual control, and often outside of the control of their community as a whole. However, these conditions also contribute to the disempowerment of these women. Poor road conditions, inability to access and afford safe transport options, as well as the burden of distance and inaccessibility of hospitals especially during the rainy season, systematically limit women’s abilities to access and utilise care at the hospital during childbirth [37]. These wider structural barriers heavily influence any decision-making processes. Women and their communities are dependent upon the Government to improve these larger structural failings, but the effectiveness of lobbying national powers will be reduced due to the limited education and power these individuals and their communities realistically hold.

### Strengths and limitations

This is the first qualitative study to be conducted after the introduction of the FHCI; however, it was conducted before the recent Ebola crisis. The influence of gender and positions of power on decision-making processes are unlikely to have changed, and structural barriers such as distance and financial obstacles will still be present, if not worsened, as the Ebola crisis has negatively impacted upon an already weak health system. When interpreting the findings, it should be noted that the main researcher was not from Sierra Leone. However, the research assistant, who was originally from the research area, was fluent in both Temne and English, and had an active role in the preliminary data analysis and adaptation of interview guides as necessary.

Regarding the use of qualitative methodologies, we consider our sampling method, which included many different groups of people and a relatively large number of respondents, along with triangulation of methods, to have strengthened both the internal and external validity of the findings. This study is qualitative and aimed at identifying findings that are of significance when planning and implementing services for these women and their communities. Through an emergent design, securing a sample of a variety of different people, representing different and relevant interests and perspectives, we believe we have identified clear patterns that are highly likely to be transferable to similar settings in rural Sierra Leone.

## Conclusions

Pregnant women in rural Sierra Leone face multiple, intersecting vulnerabilities, including poverty, gender inequality, and unequal distribution of money, power and resources. Decisions made during childbirth in rural Sierra Leone are influenced by constraints of poverty and other social determinants that are often out of the direct control of the individuals involved. The decision-making processes are complex and dynamic, with final decisions often being pragmatic depending upon which situation or circumstance women and their communities find themselves.

Gender inequality resulting in unequal power relations within households and the communities, may impact upon a woman’s ability to make fully autonomous decisions. Although efforts are being made to tackle gender inequality within Sierra Leone, true gender equality is a long way off, and men often remain highly influential in the decision-making processes either directly through ‘giving permission for treatment’ or indirectly through control of money and resources. Unfortunately men in rural Sierra Leone often lack information and understanding about childbirth, and therefore have limited knowledge on what to base their decisions, potentially resulting in harmful consequences. Approaches to fill this knowledge-gap, as well as the involvement of men in the planning and implementation of future initiatives aimed at improving maternal and infant health during pregnancy and childbirth are recommended.

A positive and significant approach towards improving access to healthcare for pregnant women has been the introduction of the FHCI. However, further steps need to be taken to ensure access includes the most vulnerable and socially marginalised, especially poor women living in isolated rural areas. Enabling women to be admitted to hospital without worrying about extra burdens and costs that using the current health system implies is necessary. Local strategies to provide families with support in childcare and other domestic and farm tasks during longer periods of pregnancy related hospitalization could be one such effort. Distance, inaccessible roads and lack of transport remain huge barriers that are often out of the direct control for many women. Community strategies such as ensuring designated members of the community are prepared and equipped to carry women, especially in emergencies, could be another strategy to explore.
