# Correction: Distance, accessibility and costs. Decision-making during childbirth in rural Sierra Leone: A qualitative study

**DOI:** 10.1371/journal.pone.0196523

**Published:** 2018-04-23

**Authors:** Laura Treacy, Håkon A. Bolkan, Mette Sagbakken

The affiliation for the first and third author is incorrect. Laura Treacy is affiliated with Nadheim Kirkens Bymisjon, Norbygata 45, 0190 Oslo, Norway. Mette Sagbakken is affiliated with Department of Nursing and Health Promotion, Faculty of Health Sciences, OsloMet—Oslo Metropolitan University, Postboks 4 St. Olavs plass 0130, Oslo, Norway.
